# Transposable Element Bm1645 is a Source of BmAGO2-associated Small RNAs that affect its expression in *Bombyx mori*

**DOI:** 10.1186/s12864-017-3598-5

**Published:** 2017-02-23

**Authors:** Hongqiang Si, Yunjie Cao, Honglin Zhu, Dan Li, Zhengbing Lv, Qing Sheng, Zuoming Nie

**Affiliations:** 1 College of Life Sciences, Zhejiang Sci-Tech University, Hangzhou, Zhejiang China; 2 Zhejiang Provincial Key Laboratory of Silkworm Bioreactor and Biomedicine, Hangzhou, Zhejiang China

**Keywords:** TEs, Bm1645, TE-siRNA, BmAGO2-associated small RNAs

## Abstract

**Background:**

A transposable element (TE) is a DNA fragment that can change its position within a genome. Transposable elements play important roles in maintaining the stability and diversity of organisms by transposition. Recent studies have shown that approximately half of the genes in *Bombyx mori* are TEs.

**Results:**

We systematically identified and analyzed the BmAGO2-associated TEs, which exceed 100 in the *B. mori* genome. Additionally, we also mapped the small RNAs associated with BmAGO2 in *B.mori.* The transposon Bm1645 is the most abundant TE associated with BmAGO2, and Bm1645-derived small RNAs represent a small RNA pool. We determined the expression patterns of several Bm1645-derived small RNAs by northern blotting, and the results showed there was differential expression of multiple small RNAs in normal and BmNPV-infected BmN cells and silkworms from various developmental stages. We confirmed that four TE-siRNAs could bind to BmAGO2 using EMSA and also validated the recognition sites of these four TE-siRNAs in Bm1645 by dual-luciferase reporter assays. Furthermore, qRT-PCR analysis revealed the overexpression of the four TE-siRNAs could downregulate the expression of Bm1645 in BmN cells, and the transcription of Bm1645 was upregulated by the downregulation of BmAGO2.

**Conclusions:**

Our results suggest Bm1645 functions as a source of small RNAs pool and this pool can produce many BmAGO2-associated small RNAs that regulate TE’s expression.

**Electronic supplementary material:**

The online version of this article (doi:10.1186/s12864-017-3598-5) contains supplementary material, which is available to authorized users.

## Background

The Argonaute (AGO) protein family contains proteins that are highly conserved throughout evolution. These proteins are the core components of the RNA-induced silencing complex (RISC) that function to silence gene expression in both plants and animals [1–4]. There are several AGO proteins present in different species. For example, eight types of AGO proteins in the human and mouse, while 27 AGO proteins in *C. elegans* [2]. There were four members of the AGO family systemically identified in *Bombyx mori* [5]. The proteins are termed BmAGO1, BmAGO2, BmAGO3, and BmPIWI. The first two members belong to the AGO subfamily, and the latter two proteins are members of the PIWI subfamily. The AGO proteins bind to small non-coding RNAs (sRNAs) to complete the RISC complex; the functions of most sRNAs are mediated through the AGO proteins.

sRNAs are important regulatory molecules in gene silencing and involved in the regulation of many cellular activities. There are three major classes of small RNAs: microRNAs (miRNAs), small interfering RNAs (siRNAs) and Piwi-interacting RNAs (piRNAs) [6]. All the three classes of small RNAs can be modulated by different mechanisms to generate various products with diverse functions. siRNAs are a special type of small RNA generated from exogenous dsRNAs that act in the process of RNA interference. Several endogenous double-stranded RNA substrates are sources of siRNAs, including long hairpin structures [7] and transposable elements [8, 9]. The endogenous siRNAs could control the activity of the TEs and regulate the expression of some genes, and they could also work for viral defense in virus-infected cells [10–17].

A TE is a DNA sequence that can change its position in a genome. TEs can be categorized into two classes (Class I and II TEs) according to their replicative and “cut and paste” mechanisms of transposition, and have important roles in maintaining the stability and diversity of organisms through transposition [18]. TEs were first discovered in plants because of their effects on genome structure and gene function [19]. The *Bombyx mori* genome contains more TE content than other insect genomes such as that of *Anopheles gambiae* genome (16%) [20] and *Tribolium castaneum* genome (33%) [21]. The TE content in *B. mori* genome was estimated to be 43.6% [22]. Thus, approximately half of the genes in the *B. mori* genome are TEs [23]. TEs can produce small RNAs including miRNAs, siRNAs, and piRNAs. A recent study in the silkworm has shown that there are 12,869 types of *Bombyx* small RNAs associated with transposons or repetitive sequences termed repeat-associated small interfering RNAs (rasiRNAs) [24]. These small RNAs may regulate transposon activity forming a genomic defense system against transposons in *Bombyx mori*. Another study identified a variety of TE-associated siRNAs (TE-siRNAs). Approximately 60% of these TE-siRNAs match the antisense strand of Bm1645 [25], which is regarded as an important source for producing small RNAs. Our group confirmed that BmAGO2 could associate with TE-siRNAs derived from several TEs, including Bm1645 [26].

In this study, we confirmed the TE Bm1645 was associated with BmAGO2 and we determined that Bm1645 is the TE with the highest abundance of TE-derived siRNAs associated with BmAGO2, which further confirms that Bm1645 was a source of small RNAs pool. Furthermore, we determined the expression profile of the Bm1645-associated TE-siRNAs associated with BmAGO2 in normal and virus-infected BmN cells and silkworms at different developmental stages. Our data indicate Bm1645-associated TE-siRNAs can bind to endogenous BmAGO2 *in vitro* and downregulate the transcription of Bm1645 in the BmN cell line. These results further support that the RNAi system can produce short interfering RNAs (siRNAs) from Bm1645 to regulate its expression.

## Results and discussion

### Sequencing and analysis of BmAGO2-associated TEs and BmAGO2-associated small RNAs

In our previous work, we confirmed that BmAGO2 could associate with TE-siRNAs derived from several TEs [26]. To determine if BmAGO2 could associate with TEs, we successfully isolated the BmAGO2-associated RNAs from BmN cells by immunoprecipitation (IP) according to the previous method [26] (Additional file 1: Figure S1) and the >200 nt fraction was size-fractionated for deep sequencing (LC Sciences). We identified 2320 TE sequences using tools for TE analysis including an online database, BmTEdb [27], and NCBI. There were 1634 BmAGO2-associated TEs in the sequences identified by the high-throughput digital expression spectrum sequencing of BmAGO2-associated RNAs. The types of these TEs include LINE, LTR, SINE, non-LTR and TIR. We identified 27 TEs with the abundance (FPKM) of more than 100, and Bm1645 was the TE most associated with BmAGO2 (FPKM = 72268.6). We obtained similar results for the small RNAs mapped. The analysis results are listed in Additional file 1: Table S1. Thus, Bm1645 was selected for further examination.

BmAGO2 is the *Bombyx* argonaute2 protein, which is the mediated protein for both the miRNA and the siRNA pathways and can bind to miRNAs or siRNAs [28–30]. As a *Bombyx* non-LTR retrotransposon, Bm1645 alone contributes to the generation of TE-associated small RNAs in a very significant way and is regarded as a source of small RNA pool [25]. The above results suggested that Bm1645 and Bm1645-associated small RNAs can load onto BmAGO2 with a high abundance. TE-associated small RNAs may regulate transposon activity forming a genomic defense system against transposons in *Bombyx mori* [24], suggesting that BmAGO2 and Bm1645-associated small RNAs could be involved in the maintenance of genome stability by suppressing the activity of Bm1645.

### Analysis of the expression differences for several TE-siRNAs in normal and virus-infected silkworm

**Fig. 1:**
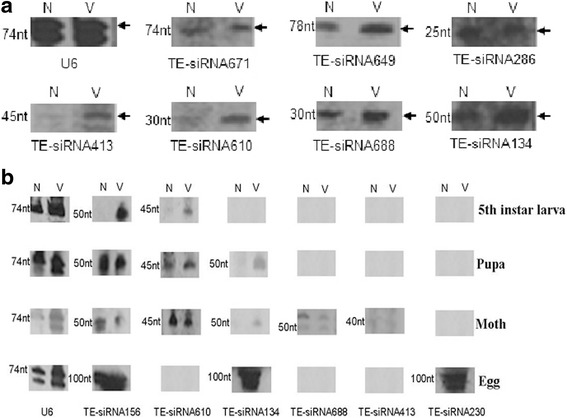
Identification of TE-siRNAs by northern blotting in BmN cells and each phase of the silkworm. **a** Identification of TE-siRNAs by northern blotting in BmN cells. N: normal BmN cells, V: BmNPV-infected BmN cells. There were 7 of 13 TE-siRNAs detected in BmN cells. The detected TE-siRNAs were TE-siRNA134, TE-siRNA286, TE-siRNA413, TE-siRNA610, TE-siRNA649, TE-siRNA671 and TE-siRNA688. We used U6 as the control. There were obvious differences of the expression of TE-siRNA413 and TE-siRNA610 between the normal and virus-infected BmN cells. **b** Expression profile of TE-siRNAs in each phase of the silkworm by northern blotting. N: normal samples, V: BmNPV-infected samples. Only TE-siRNA156 could be detected in all four developmental stages of the *Bombyx mori*. The others were expressed in one, two, or three different stages

Baculovirus is an important virus for silkworm and it is the pathogen of silkworm nuclear polyhedrosis. Virus infection can trigger widespread silencing of host genes by endogenous siRNAs [16]. We wanted to know if there is a correlation between TE-siRNAs and the baculovirus infection in *Bombyx mori*. Therefore, we performed northern blotting to further test the expression of TE-siRNAs in normal and ie1-bacmid-pIEx-1-BmAGO2-infected BmN cells and silkworms at different developmental stages. We chose 13 BmAGO2-associated TE-siRNAs derived from Bm1645 according to the previous analysis and then synthesized probes. U6 was used as the control. In normal and virus-infected BmN cells, we detected the following TE-siRNAs by northern blotting: TE-siRNA134, TE-siRNA286, TE-siRNA413, TE-siRNA610, TE-siRNA649, TE-siRNA671 and TE-siRNA688. We found there were obvious differences in the expression of TE-siRNA413 and TE-siRNA610 between the normal and virus-infected BmN cells (Fig. 1a). Unexpectedly, a set of SINE-derived endo-siRNAs was also found in a variety of sequencing data and MHV68-infected cells in mouse [31]. Sindbis virus (SINV) could also induce the production of another class of mRNA-derived endo-siRNAs in *Aedes aegypti* [32]. This finding suggests there is a correlation between these TE-siRNAs and the baculovirus infection in *Bombyx mori*. The activation of antiviral RNAi in silkworm might be accompanied by these endogenous TE-siRNAs. The specific functions require further research.

The expression of the TE-siRNAs was different during different developmental stages of the virus-infected silkworms compared to the normal individuals. Figure 1b shows that only TE-siRNA156 was detected in all four developmental stages of *Bombyx mori*. The other TE-siRNAs were expressed in one, two, or three different stages. The TE-siRNAs were selectively expressed in different developmental stages. The results suggest the TE-siRNAs identified do not exist during the entire life stages of the mulberry silkworm. We found TE-siRNA156, TE-siRNA610, and TE-siRNA134 were expressed in most of the life stages. Furthermore, TE-siRNA688, and TE-siRNA413 were detected only in moths, and TE-siRNA230 was detected only in eggs. There were several TE-siRNAs identified whose expression was affected by the virus, including TE-siRNA134 and TE-siRNA610. However, the expression of most TE-siRNAs remained relatively constant between normal and virus-infected silkworm.

### TE-siRNAs combine with activated BmAGO2 *in vitro*

We performed EMSA (Electrophoretic Mobility Shift Assay) to further identify the interaction between TE-siRNAs and BmAGO2. This assay is often used to detect interaction between DNA/RNA and proteins [33, 34]. The EMSA technique is based on the principle that the rate of nucleic acid migration is slowed when bound to protein.

**Fig. 2:**
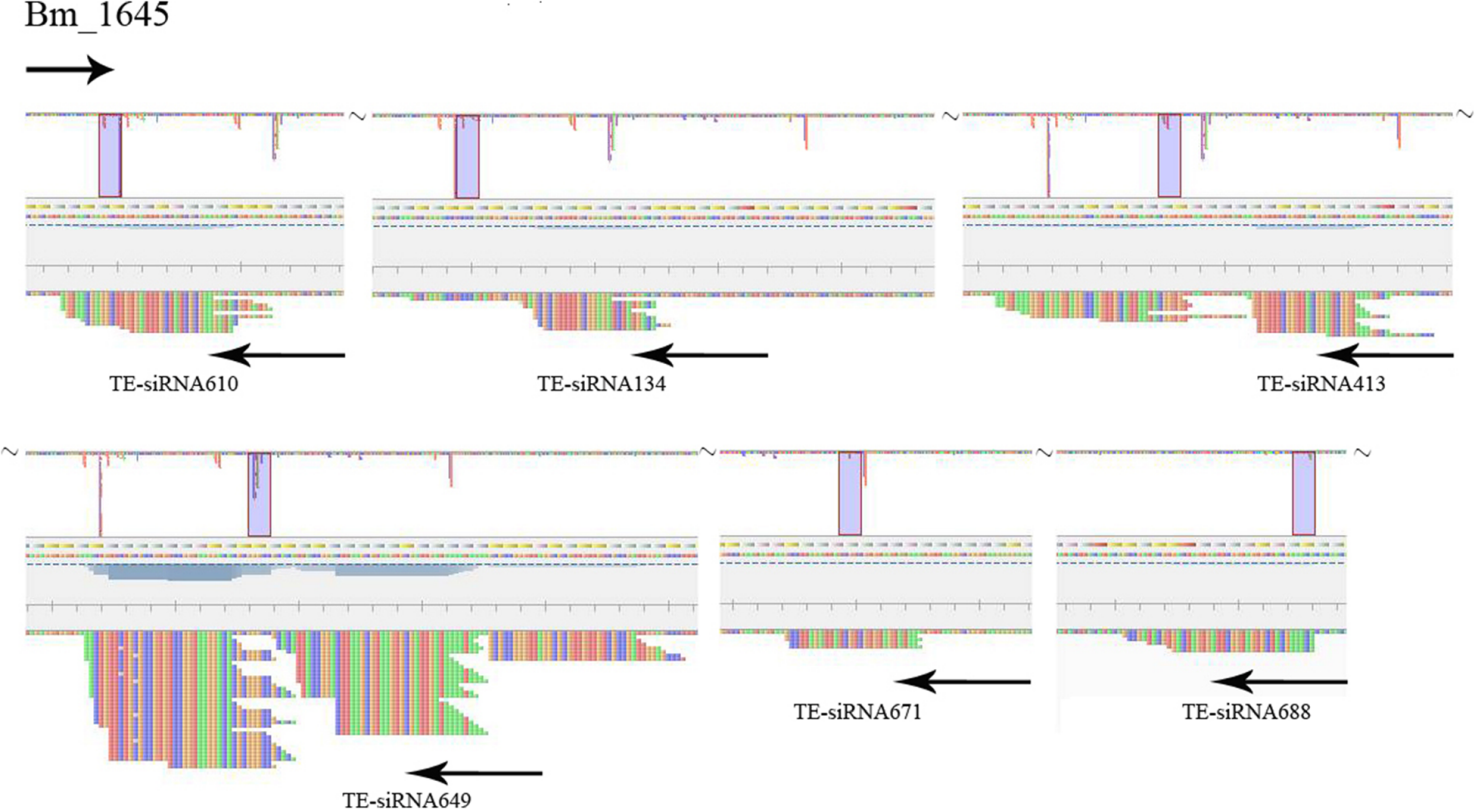
The mapping pattern of Bm1645 and 6 Bm1645-derived small RNAs. These 6 TE-siRNAs are reversely complementary to Bm1645 and show relatively high abundance. We chose these as our targets for subsequent experiments. The mapping figure was generated using the Tablet software

**Fig. 3:**
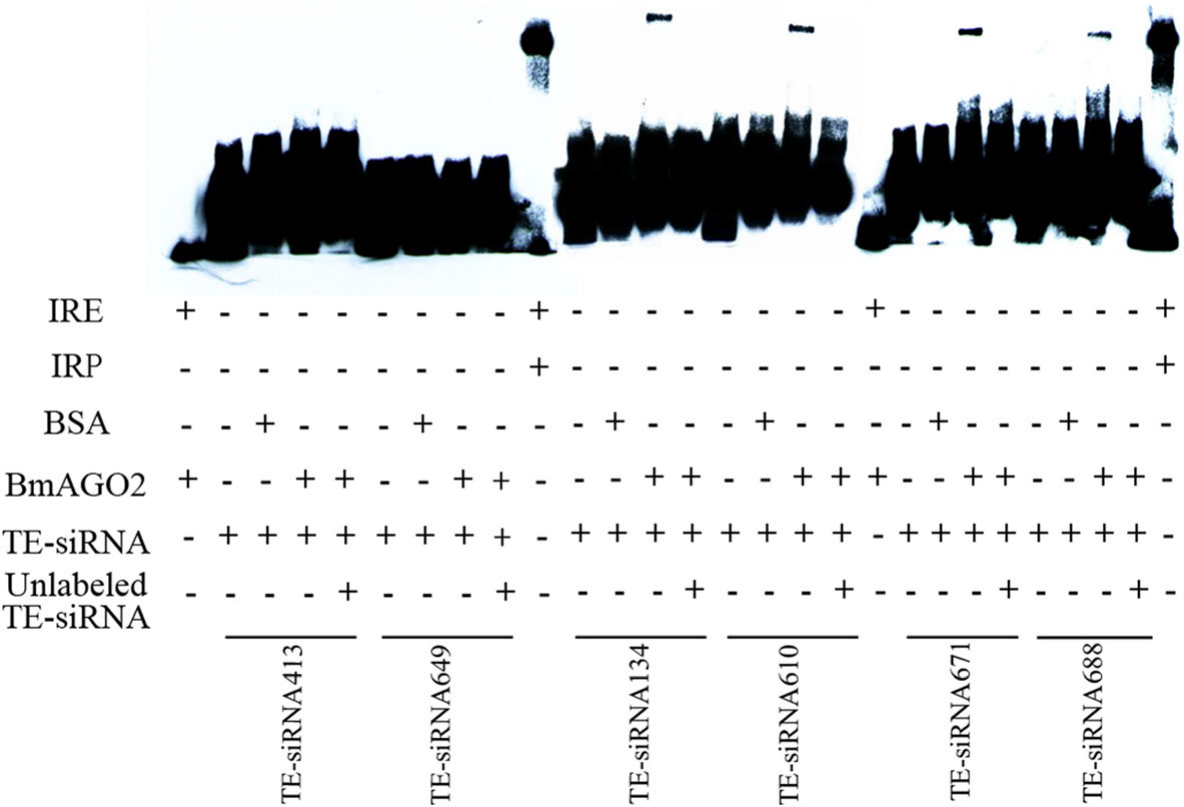
TE-siRNAs combine with activated BmAGO2 *in vitro*. We used the Control IRE/IRP System included in the kit as one positive control and the IRE/BmAGO2 and TE-siRNAs without proteins as the negative control. The shift of the positive control was a thick stripe. We detected 6 TE-siRNAs, and there were 4 detected with block stripes. The shifted TE-siRNAs were TE-siRNA134, TE-siRNA610, TE-siRNA671, and TE-siRNA688. On the contrary, TE-siRNA413 and TE-siRNA649 were not associated with BmAGO2

We chose 6 TE-siRNAs from the pool used in the previous experiment that were reverse complements of Bm1645 and in relatively high abundance (Fig. 2). The results suggested that 4 of the 6 TE-siRNAs were identified to associate with BmAGO2. The associated TE-siRNAs were TE-siRNA134, TE-siRNA610, TE-siRNA671, and TE-siRNA688. These BmAGO2-associated TE-siRNAs migrate more slowly than the corresponding free TE-siRNAs (Fig. 3). Conversely, TE-siRNA413 and TE-siRNA649 were not identified to associate with BmAGO2 *in vitro*, implying there might be another factor that affect the association between the two TE-siRNAs and BmAGO2 *in vivo*. The experiment *in vitro* simulated the combination of the TE-siRNAs and BmAGO2. This result reveals that the small RNAs generated by Bm1645 (TE) associate with activated BmAGO2, and suggests that Bm1645 TE-siRNAs might act to regulate Bm1645 expression *in vivo*.

### TE-siRNAs downregulate the expression of Bm1645 *in vitro*

**Fig. 4:**
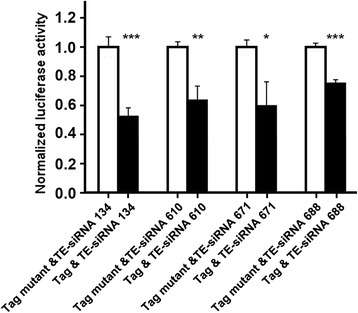
Validation of TE-siRNA targeted sites using dual-luciferase assays. The expression of the firefly luciferase fused with the wild-type sequences containing TE-siRNA targeted sites in Bm1645 was inhibited by the corresponding TE-siRNAs. But TE-siRNAs did not affect the activity of luciferase fused with the mutant sequences

**Fig. 5:**
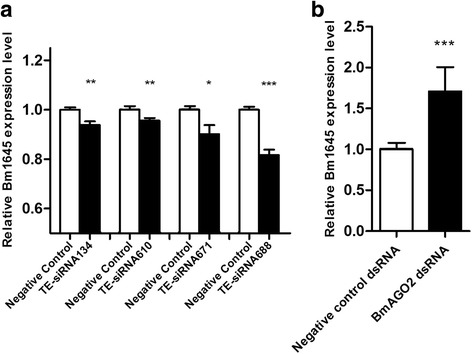
The relative expression level of Bm1645. **a** The qRT-PCR analysis of Bm1645 in TE-siRNAs-transfected BmN cells. The expression was downregulated when the BmN cells were transfected with TE-siRNAs. A negative control was used as contrast. **b** The transcription level of Bm1645 affected by BmAGO2 knockdown in BmN cells. We used the RNA interference method to downregulate the expression of BmAGO2. The result shows the transcriptional level of Bm1645 was upregulated 1.66 times, as assessed by qRT-PCR. Each histogram bar represents the mean relative expression of the indicated transcripts, with at least three replicates for each bar. The asterisks (*, ** or ***) indicate the significant differences (*P* < 0.05, *P* < 0.01 or *P* < 0.001, respectively) compared with the relevant control with a two-tailed *t*-test

We chose the 4 TE-siRNAs that associated with BmAGO2 in the EMSAs to use for the dual-luciferase reporter assays and qRT-PCR experiments with BmN cells. The results showed the 4 TE-siRNAs significantly inhibited the expression of the luciferase fused with the wild-type region containing TE-siRNA targeted sites in Bm1645 but did not affect the activity of luciferase fused with the mutant sequences (Fig. 4). These results suggest that TE-siRNAs target the targeted sites in Bm1645 to suppress the gene’s expression. Furthermore, the transcription of Bm1645 was downregulated in BmN cells transfected with TE-siRNAs compared with the negative control (Fig. 5a). When the expression of BmAGO2 was downregulated by RNA interference, the transcriptional level of Bm1645 was upregulated 1.66 times, as assessed by qRT-PCR (Fig. 5b). These results suggest that there is a relationship between BmAGO2 and Bm1645 in BmN cells and the bridge might be the small RNAs. BmN is a *Bombyx* ovary cell line. In many reports, endo-siRNAs are always involved in the germline development [35–38]. The regulatory function by TE-siRNAs in BmN cells suggests a relationship between these siRNAs and germline in silkworm.

There are three types of small RNAs and researchers have searched for transposable elements that bind these small RNAs. Previous studies found that piRNAs repressed TEs in the animal germ line [39]. There are differences between piRNAs and miRNAs/siRNAs; piRNAs bind to the Piwi class of Argonaute proteins, however, miRNAs/siRNAs bind to the Ago class of Argonaute proteins [39]. One study [40] expanded the scope of investigated species [17, 41] from two strains of *D. melanogaster* to 16 strains and discovered there was novel piRNA production at novel TE loci. These findings were consistent with data reported in previous studies [17, 42]. Another report found a relationship between piRNAs and TEs [43]. The report suggested the movement of TEs was repressed by piRNAs in the gonadal cells of *Drosophila*. One of the inhibition mechanisms was through the formation of heterochromatin and then contact with repressed gene expression [44]. A recent study has found there is another method used to repress TEs, which is endo-siRNA inhibition [14]. In addition to insects, there were related reports in plants describing the relationship between small RNAs and TEs [45]. Although there have been many discoveries involving TEs and piRNAs, there is less relevant research with siRNAs and TEs in insects, such as the silkworm. RNA interference is a useful tool for the downregulation of target gene expression [46]. There is insufficient information describing other roles for siRNAs except for those known in insects. Thus, plants are commonly used to study TEs with siRNAs. Plants produce 24-nt small interfering RNAs that are mainly derived from repeats and TEs. For example, rice uses Dicer-like 3 to produce TE-associated 24-nt siRNAs to control agricultural traits [47]. There are a limited number of studies about TEs and siRNAs in *Bombyx mori*. Based on our previous research [26], we systematically identified and analyzed the TEs associated with BmAGO2 that had an abundance value (FPKM) exceeding 100. We also analyzed their mapped small RNAs associated with BmAGO2. We found that Bm1645 was the most abundant BmAGO2-associated TE, and the abundance of its mapped small RNAs was also the most. These results matched with our previous study [26]. We chose Bm1645 and its siRNAs as our targets and identified their interactions. Our results showed TE (Bm1645)-siRNAs could associate with activated BmAGO2 *in vitro*. This finding suggested Bm1645 could produce siRNAs that were also associated with BmAGO2 *in vivo*. We hypothesize that Bm1645 might regulate its own expression with the help of the siRNAs. We also validated the targeted sites of TE-siRNAs in Bm1645 and found the upregulation of both the siRNAs and BmAGO2 influenced the relative expression of Bm1645. The results confirm our hypothesis, suggesting there is a bridge between BmAGO2 and Bm1645 which might be the siRNAs. Bm1645 is a BmAGO2-associated TE that possibly plays an important role in cell growth. It is also regarded as a small RNA pool [25] and may serve as a siRNAs source acting to silence its own activity. BmAGO2 could combine with Bm1645 to promote the function of TEs in gene regulation by TE-siRNAs. In *Bombyx mori*, there may be a second way to inhibit TEs activity with the help of the siRNAs in addition to the piRNA pathway.

## Conclusions

We found Bm1645 was the most abundant BmAGO2-associated TE, and Bm1645-derived small RNAs was also the most abundant BmAGO2-associated small RNAs, suggesting Bm1645 was a source of small RNAs pool. We determined that several Bm1645-derived small RNAs showed differential expression in normal and BmNPV-infected BmN cells and silkworms from various developmental stages. Furthermore, we found four TE-siRNAs from Bm1645 could bind to endogenous BmAGO2 and downregulate the expression of Bm1645 in BmN cells, suggesting Bm1645 may serve as a siRNAs source acting to regulate its own expression.

## Methods

### RNA-Seq and data analysis

The BmAGO2-associated RNAs were size-fractionated for the >200 nt fraction by a polyacrylamide gel electrophoresis according to the previous work [26]. The >200 nt fraction was subjected to library construction and deep sequencing using Illumina Hiseq 2000 following the vendor’s recommended protocol. Adaptors, low-quality tags and contaminants were removed from raw reads to produce the clean total reads. Totally, the high throughput sequencing yielded a total of 11,528,900 reads with a 36 bp read length (SRA: SRR5136808). RSEM [48] was used to estimate the expression levels of all TEs. As an appropriate software tool for estimating the expression of repeated sequences, such as TEs [49], RSEM calculate TPM and FPKM values for the measurement of gene expression levels. We used bowtie software [50] to map TE-siRNAs (GEO: GSM1025527) to the TEs with the FPKM of more than 100.

### Insects and cell lines

Silkworm larvae were raised on fresh mulberry leaves at 27 °C in 75–80% humidity in an artificial climate incubator. The BmN cell line was cultured in Sf-900 II serum-free medium (Gibco BRL) with 10% (v/v) fetal bovine serum (Gibco BRL) at 27 °C. The cells preserved in our lab were derived from the silkworm ovary.

### RNA transfection

TE-siRNA134, TE-siRNA413, TE-siRNA610, TE-siRNA649, TE-siRNA671, TE-siRNA688 and the negative control were synthesized by Genepharma (Shanghai). The BmN cells were transfected with 5 μg TE-siRNA and negative control mixed with 10 μl X-tremeGENE siRNA Transfection Reagent (Roche) in Sf-900 II SFM at a density of 40–50% in each well of a 6-well plate. RNA extraction and reverse transcription were performed according to the manufacturer’s protocol 72 h after transfection,. The experiment was performed with three technical repeats.

We used the T7 RiboMAX™ Express RNAi System Kit (Promega) to synthesize the BmAGO2 dsRNA according to the protocol. The negative control dsRNA was synthesized by Genepharma (Shanghai). The transfection process was the same as the TE-siRNAs transfection.

### Expression and purification of the BmAGO2

We collected a number of fifth instar larvae, pupae and second day moths and inoculated the ie1-bacmid–pIEx-1-BmAGO2 that was previously constructed and preserved in our lab [51]. BmAGO2 was expressed using the Bacmid system. We also collected the eggs and used the normal individuals as a control. After infection, we used liquid nitrogen for sample grinding and then added the TRIzol reagent (Invitrogen). The BmN cells were infected with the ie1-bacmid–pIEx-1-BmAGO2 when the density was 80%, and the infected cells were cultivated in an incubator at 27 °C until they had obvious infection symptoms. Normal cells were used as a control.

We purified the BmAGO2 protein by centrifuging to collect the cells 72 h after viral infection. The cells were lysed using lysis buffer for 30 min on ice, and then ultrasonication was performed for 15 s three times. The lysate was centrifuged at 12000 rpm for 15 min. The supernatant was mixed with injector and then added to the corresponding Nickel beads (Millipore) according to the protocol. After washing three times with washing buffer, we added 100 μl of eluent buffer to dissolve the BmAGO2 protein.

### RNA extraction

The total RNA was extracted by using the Direct-zol™ RNA MiniPrep Kit (Zymo Reasearch) according to the manufacturer’s protocol. We collected RNA from silkworm at different developmental stages ranging from eggs to moths, normal and infected BmN cells, and cells that were transfected with TE-siRNAs for approximately 72 h, respectively. We determined the RNA concentration by using the Nanodrop ND-100 spectrophotometer.

### Northern blotting

RNA was separated using 15% denaturing polyacrylamide gel electrophoresis (PAGE) containing urea. After electrophoresis, the RNA was transferred to a nylon membrane (Millipore) for the northern blotting experiment. We used the DIG Oligonucleotide Tailing Kit (Roche) to label the probes synthesized by Sangon Biotech (Shanghai). Illustra ProbeQuant G-25 Micro Columns (GE Healthcare) were used for the purification of the probes after labeling. The northern blotting was performed for normal and infected individuals of the following stages: fifth instar larvae, pupae, moths, and eggs. The membrane with the RNA was then subjected to UV cross-linking, pre-hybridized at 40 °C for 2 h, followed by 16 h hybridization with probes at 37 °C. Next, the membrane was washed twice at room temperature for 25 min using 2 × SSC and 0.1% SDS and twice at 37 °C using 0.5 × SSC and 0.1% SDS with gentle shaking. The detection experiment was performed using the DIG Luminescent Detection Kit (Roche) according to the manufacturer’s protocol.

### Quantitative real-time PCR (qRT-PCR)

The total RNA extracted from the transfected BmN cells was reverse transcribed to cDNA using the Transcriptor First Strand cDNA Synthesis Kit (Roche) according to the manufacturer’s procedures. The random primers were used for the reaction. FastStart Universal SYBR Green Master (Rox) (Roche) was used for the qRT-PCR reactions, which were performed using the following the protocol: 95 °C for 10 min, followed by 40 cycles of 95 °C for 15 s and 60 °C for 1 min, 95 °C for 15 s, 60 °C for 1 min and 95 °C for 15 s. The experiment was performed three times with three replicates and normalized to the control. β-actin was the internal control for the Bm1645 mRNA.

### Electrophoretic Mobility Shift Assay (EMSA)

The synthesized TE-siRNAs were labeled with a single biotinylated nucleotide to the 3’ terminus of one strand using the Pierce™ RNA 3’ End Biotinylation Kit (Thermo). The EMSA and signal detection were performed using the LightShift Chemiluminescent RNA EMSA Kit (Thermo) and Chemiluminescent Nucleic Acid Detection Module (Thermo) according to the manufacturer’s protocol. We used the Control IRE/IRP System included in the kit as a positive control and the IRE/BmAGO2 and TE-siRNAs without protein as the negative control.

### Dual-luciferase reporter assays

The wild-type region of Bm1645 containing TE-siRNA targeted sites and mutant region were synthesized and cloned into the dual-luciferase reporter vector pIEx-1-Rluc-Luc constructed by our laboratory. The wild-type and mutant sequences used in reporter vector construction are listed in Additional file 1: Table S2. BmN cells were plated on 24-well plates and were co-transfected with 300 ng reporter plasmid and 500 ng TE-siRNA. Cells were lysed 24 h after transfection and then luciferase activities were measured by Dual-Luciferase® Reporter Assay System (promega) according to the manufacturer’s instructions. Firefly luciferase expression was normalized by the expression of renilla luciferase. Three biological replicates were performed.

## Additional file

Additional file 1: Table S1. The BmAGO2-associated TEs with abundance more than 100 and their mapped BmAGO2-associated small RNAs*. **Table S2.** The wild-type sequences of Bm1645 containing TE-siRNA targeted site and mutant sequences for dual-luciferase reporter vector construction. **Figure S1.** (A) Western blotting analysis of HIS-BmAGO2 isolated by immunoprecipitation. BmAGO2 was expressed in BmN cells infected with recombinant BmNPV virus. IP was validated by western blotting. (B) Co-immunoprecipitation RNAs analyzed by electrophoresis. M: marker. 1: RNAs pulled down by HIS monoclonal antibody. 2: RNAs pulled down by mouse IgG. The lane 2 was the negative control. Asterisks indicate the visible bands for the ~20 nt, ~27 nt and ~33 nt small RNA species, respectively. The box in lane 1 indicate the >200 nt fraction extracted for deep sequencing. (DOC 137 kb)

## Abbreviations

AGO: Argonaute
EMSA: Electrophoretic mobility shift assay
IP: Immunoprecipitation
PAGE: Polyacrylamide gel electrophoresis
piRNAs: Piwi-interacting RNAs
qRT-PCR: Quantitative real-time PCR
rasiRNAs: repeat-associated small interfering RNAs
RISC: RNA-induced silencing complex
siRNAs: small interfering RNAs
sRNAs: small non-coding RNAs
TE: Transposable element
TE-siRNAs: TE-associated siRNAs
