# Supplementary material for: Transposable Element Bm1645 is a Source of BmAGO2-associated Small RNAs that affect its expression in Bombyx mori
Source: BMC Genomics. 2017 Feb 23;18:201. doi: 10.1186/s12864-017-3598-5 (PMC5324241; doi:10.1186/s12864-017-3598-5)
Supplement: Additional file 1: Table S1. — The BmAGO2-associated TEs with abundance more than 100 and their mapped BmAGO2-associated small RNAs*. Table S2. The wild-type sequences of Bm1645 containing TE-siRNA targeted site and mutant sequences for dual-luciferase reporter vector construction. Figure S1. (A) Western blotting analysis of HIS-BmAGO2 isolated by immunoprecipitation. BmAGO2 was expressed in BmN cells infected with recombinant BmNPV virus. IP was validated by western blotting. (B) Co-immunoprecipitation RNAs analyzed by electrophoresis. M: marker. 1: RNAs pulled down by HIS monoclonal antibody. 2: RNAs pulled down by mouse IgG. The lane 2 was the negative control. Asterisks indicate the visible bands for the ~20 nt, ~27 nt and ~33 nt small RNA species, respectively. The box in lane 1 indicate the >200 nt fraction extracted for deep sequencing. (DOC 137 kb) [file 12864_2017_3598_MOESM1_ESM.doc]

**SUPPLEMENTARY MATERIALS**

**Table S1** The BmAGO2-associated TEs with abundance more than 100 and their mapped BmAGO2-associated small RNAs*

| **Name of TEs** | **Types of TEs** | **Length（bp）** | **The abundance of TEs** **associated with BmAGO2 (****FPKM)** | **The abundance of mapped small RNAs associated with BmAGO2** |
| --- | --- | --- | --- | --- |
| Bm1645 | LINE/R4 | 13595 | 72268.9 | 226535 |
| Bm640 | Unknown/Unknown | 517 | 5076.6 | 40 |
| Bm2125 | Unknown/Unknown | 204 | 2725.1 | 4 |
| Bm1837 | Unknown/Unknown | 115 | 1020.9 | 12 |
| Bm1921 | Unknown/Unknown | 108 | 598.4 | 0 |
| Bm1228 | SINE/Bm1 | 123 | 516.9 | 58 |
| Bm1109 | SINE/Unknown | 165 | 381.2 | 232 |
| Bm1970 | Unknown/Unknown | 610 | 335.9 | 1 |
| Bm1222 | Unknown/Unknown | 1468 | 332.5 | 7563 |
| M19755 | LINE/R1 | 5243 | 272.3 | 1739 |
| Bm1236 | SINE/Bm1 | 165 | 262.9 | 48 |
| AB076841 | Unknown/Unknown | 4372 | 252.6 | 632 |
| AB032718 | SINE/Bm1 | 443 | 235.7 | 1877 |
| Bm1867 | SINE/Unknown | 107 | 202.9 | 6 |
| BM1B | SINE/Unknown | 177 | 197.3 | 179 |
| Bm2033 | LTR/Unknown | 289 | 190.5 | 2 |
| Bm979 | Unknown/Unknown | 672 | 187.9 | 34 |
| Bm2216 | LTR/Unknown | 362 | 183.7 | 2 |
| Bm2096 | SINE/Bm1 | 124 | 142.3 | 22 |
| Bm767 | DNA/Tc1_mariner | 964 | 136.8 | 2555 |
| BM1 | SINE/BM1 | 445 | 132.5 | 382 |
| Bm870 | Unknown/Unknown | 339 | 131.7 | 329 |
| M16558 | LINE/R2 | 4400 | 125.4 | 1227 |
| Bm1679 | SINE/Bm1 | 135 | 119.9 | 37 |
| Bm1845 | SINE/Bm1 | 163 | 117.5 | 26 |
| Bm1582 | Unknown/Unknown | 194 | 115.8 | 5 |
| Bmori_326.1597 | LINE/Unknown | 576 | 104.4 | 23581 |

*The high-throughput expression spectrum sequencing of BmAGO2-associated RNAs was performed by LC Sciences company in Hangzhou using a standard high-throughput sequencing protocol.

**Table S2** The wild-type sequences of Bm1645 containing TE-siRNA targeted site and mutant sequences for dual-luciferase reporter vector construction

| Names of TE-siRNAs | Sequences of TE-siRNAs | The wild-type sequences of Bm1645 containing TE-siRNA targeted sites* | The mutant sequences** |
| --- | --- | --- | --- |
| TE-siRNA134 | TATGCTCACTGTGGAGCCCCGAGAG | TAGAGCCTCCCGACTCTCGGGGCTCCACAGTGAGCATATCCTTGCCGGA | TAGAGCCTCCCGATATGCTCACTGTGGAGCCCCGAGAGTCCTTGCCGGA |
| TE-siRNA610 | TTGAGACTCAGCCCTGCGCCAGGTGATTCGT | GACGAATCACCTGGCGCAGGGCTGAGTCTCAACAGATCGCAGCACGACG | GTTGAGACTCAGCCCTGCGCCAGGTGATTCGTCAGATCGCAGCACGACG |
| TE-siRNA671 | TTCTCGTCGCGACGACTCCTGTCCAAG | TCAAGACGAACTTGGACAGGAGTCGTCGCGACGAGAAAGCGGAACCTCC | TCAAGACGAATTCTCGTCGCGACGACTCCTGTCCAAGAGCGGAACCTCC |
| TE-siRNA688 | TATTTGATCAGCGTCGGACCTGCGTCATG | AGATACTTCAGATCGCATGACGCAGGTCCGACGCTGATCAAATATATAA | AGATACTTCAGATCGTATTTGATCAGCGTCGGACCTGCGTCATGTATAA |

*The TE-siRNA targeted sites in the wild-type sequences of Bm1645 were underlined and these wild-type sequences were cloned into the multiple cloning site (*Sac* I/*Xho* I) located downstream of the luciferase translational stop codon in the dual-luciferase reporter vector pIEx-1-Rluc-Luc constructed by our laboratory.

**As the control, the TE-siRNA targeted sites were mutated in the wild-type sequences of Bm1645.


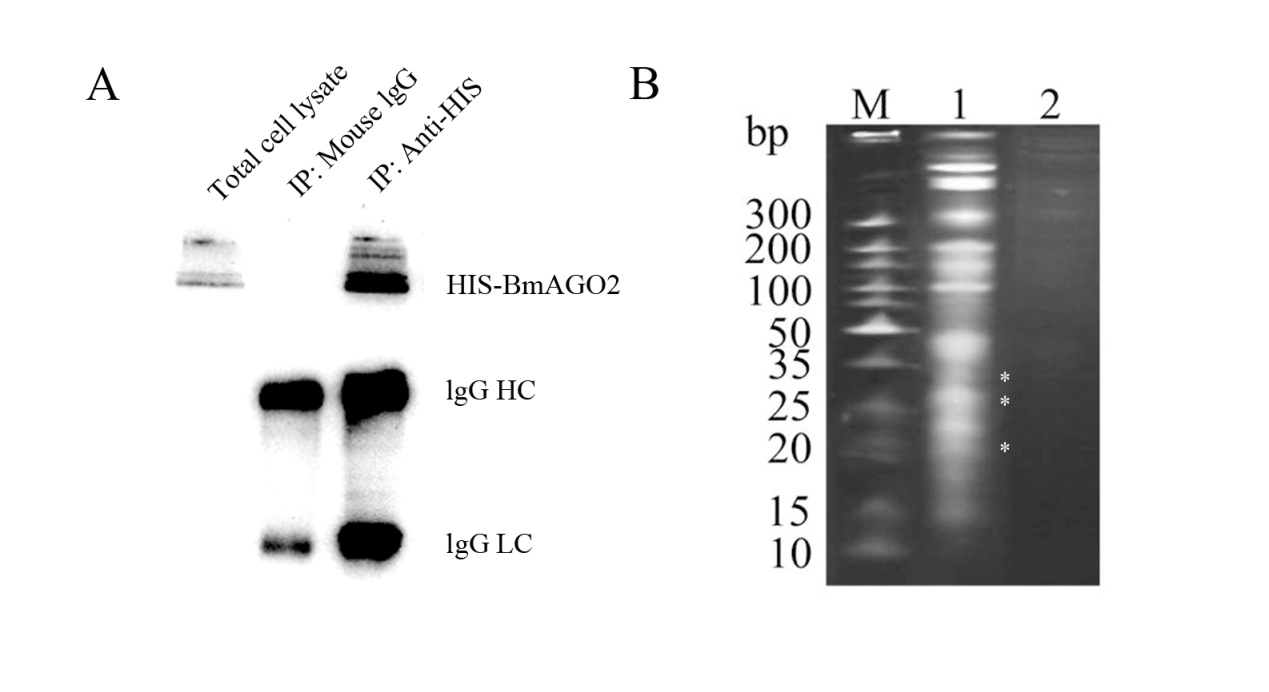


**Figure S1** (A) Western blotting analysis of HIS-BmAGO2 isolated by immunoprecipitation. BmAGO2 was expressed in BmN cells infected with recombinant BmNPV virus. IP was validated by western blotting . (B) Co-immunoprecipitation RNAs analyzed by electrophoresis. M: marker. 1: RNAs pulled down by HIS monoclonal antibody. 2: RNAs pulled down by mouse IgG. The lane 2 was the negative control. Asterisks indicate the visible bands for the ～20nt, ～27nt and ～33nt small RNA species, respectively. The box in lane 1 indicate the >200nt fraction extracted for deep sequencing.
